# Bis(5-phenyl-1*H*-1,2,4-triazol-3-yl) disulfide dihydrate

**DOI:** 10.1107/S1600536811014607

**Published:** 2011-04-29

**Authors:** Ai-Xin Zhu, Jun-Na Liu, Zhen Li, Hong-Can Wang, Yuan-Chao Du

**Affiliations:** a Faculty of Chemistry and Chemical Engineering, Yunnan Normal University, Kunming 650092, People’s Republic of China; b School of Chemical Engineering, Henan University of Science and Technology, Luoyang 471003, People’s Republic of China

## Abstract

A crystallographic twofold axis passing through the centre of the disulfide linkage in the title compound, C_16_H_12_N_6_S_2_·2H_2_O, results in one-half of the mol­ecule and one uncoordinated water mol­ecule described in the asymmetric unit. In the mol­ecule, the mean planes of the benzene and triazole rings are close to being coplanar and are separated by a dihedral angle of 2.08 (15)°. The triazole rings are twisted by a dihedral angle of 37.67 (6)° from the disulfide linkage. The crystal packing is stabilized by inter­molecular N—H⋯O and O—H⋯N hydrogen bonds with the water mol­ecules, forming a three-dimensional supra­molecular network.

## Related literature

For applications of 1,2,4-triazole and its derivatives in coordination chemistry, see: Zhang *et al.* (2005 ▶); Ouellette *et al.* (2007 ▶); Zhu *et al.* (2009 ▶). For the related structure of a 1,2,4-triazole-based disulfide compound, see: Jiang *et al.* (2007 ▶). For the previous synthesis of the title compound, see: El-Wareth & Sarhan (2000 ▶).
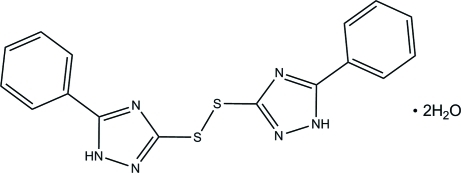


## Experimental

### 

#### Crystal data

C_16_H_12_N_6_S_2_·2H_2_O
*M* *_r_* = 388.47
Monoclinic, 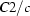

*a* = 12.3911 (13) Å
*b* = 14.7125 (16) Å
*c* = 10.2966 (11) Å
β = 104.125 (2)°
*V* = 1820.4 (3) Å^3^
*Z* = 4
Mo *K*α radiation
μ = 0.32 mm^−1^
*T* = 293 K
0.40 × 0.20 × 0.18 mm

#### Data collection

Bruker SMART APEX CCD diffractometer
Absorption correction: multi-scan (*SADABS*; Sheldrick, 1996 ▶) *T*_min_ = 0.884, *T*_max_ = 0.945
7210 measured reflections
1953 independent reflections
1679 reflections with *I* > 2σ(*I*)
*R*_int_ = 0.023

#### Refinement

*R*[*F* ^2^ > 2σ(*F* ^2^)] = 0.042
*wR*(*F* ^2^) = 0.120
*S* = 1.06
1953 reflections
118 parameters
H-atom parameters constrained
Δρ_max_ = 0.20 e Å^−3^
Δρ_min_ = −0.18 e Å^−3^

### 

Data collection: *SMART* (Bruker, 2004 ▶); cell refinement: *SAINT* (Bruker, 2004 ▶); data reduction: *SAINT*; program(s) used to solve structure: *SHELXS97* (Sheldrick, 2008 ▶); program(s) used to refine structure: *SHELXL97* (Sheldrick, 2008 ▶); molecular graphics: *DIAMOND* (Brandenburg, 1999 ▶); software used to prepare material for publication: *SHELXTL* (Sheldrick, 2008 ▶).

## Supplementary Material

Crystal structure: contains datablocks I, global. DOI: 10.1107/S1600536811014607/jj2087sup1.cif

Supplementary material file. DOI: 10.1107/S1600536811014607/jj2087Isup2.cdx

Structure factors: contains datablocks I. DOI: 10.1107/S1600536811014607/jj2087Isup3.hkl

Additional supplementary materials:  crystallographic information; 3D view; checkCIF report

## Figures and Tables

**Table 1:** Hydrogen-bond geometry (Å, °)

*D*—H⋯*A*	*D*—H	H⋯*A*	*D*⋯*A*	*D*—H⋯*A*
O1—H1*C*⋯N3	0.90	2.02	2.9210 (19)	178
N1—H1*B*⋯O1^i^	0.86	1.90	2.7077 (19)	156
O1—H1*D*⋯N2^ii^	0.84	2.07	2.909 (2)	171

Symmetry codes: (i) 
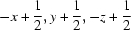
; (ii) 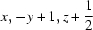.

**Fig. 1.**
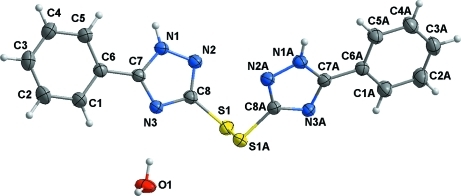
Molecular structure of the title compound, with atom labels and 30% probability displacement ellipsoids for non-H atoms.

**Fig. 2.**
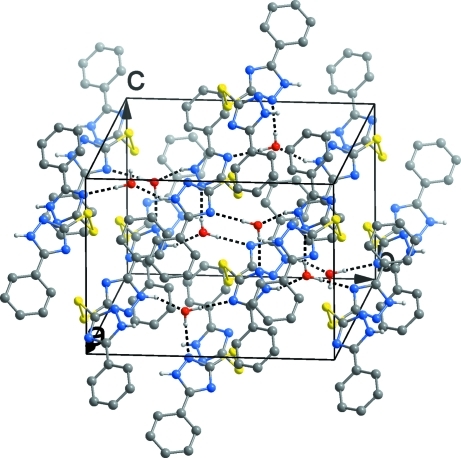
Packing diagram of the title compound viewed down the a axis. O—H···N and N—H···O hydrogen bonds with the water molecule are shown with dashed lines.

## supplementary crystallographic information

### Comment

In the past few years, 1,2,4-triazole and its derivatives have attracted increasing attention as an N-heterocyclic aromatic ligand, since they can combine both imidazoles and pyrazoles in their coordination geometry. In addition, metal-triazolate frameworks can exhibit special luminescent, magnetic and favourable gas-adsorption abilities (Ouellette *et al.*, 2007; Zhang *et al.*, 2005; Zhu *et al.*, 2009). 1,2,4-triazole based thiols and disulfides are important 1,2,4-triazole derivatives, and may exhibit a more diverse coordination geometry by combining heterocyclic nitrogen and sulfur donor atoms, and therefore affect biological activity behaviour. However, only one example of a crystallographic study on organic 1,2,4-triazole based disulfide compounds is found in the literature (Jiang *et al.* 2007). Although the synthesis of the compound 1,2-bis(5-phenyl-1*H*-1,2,4-triazol-3-yl)disulfide has been reported by El-Wareth & Sarhan (2000), no crystallographic study has been reported on the ligand and related metal coordination compounds. We reported herein another synthetic method and the crystal structure of the title compound.

A crystallographic 2-fold axis passing through the centroid of the disulfide linkage in the title compound, C_16_H_12_N_6_S_2_.2H_2_O, results in one-half of the molecule and one uncoordinated water molecule described in the asymmetric unit (Fig. 1). In the molecule, the mean planes of the benzene and triazole rings are close to coplanar, separated by a dihedral angle of 2.08 (15)°. The triazole rings are twisted by a dihedral angle of 37.67 (6)° from the disulfide linkage. Crystal packing is stabilized by intermolecular N—H···O and O—H···N hydrogen bonds with the water molecules forming a three-dimensional supramolecular network (Fig. 2).

### Experimental

A mixture of iron dichloride tetrahydrate (40 mg, 0.2 mmol), 3-phenyl-1*H*-1,2,4-triazole-5(4*H*)-thione (35 mg, 0.2 mmol), 8 ml methanol and 4 ml acetonitrile was stirred for 10 min, then filtered and allowed to stand at room temperature for about two weeks. Yellow polyhedron crystals suitable for X-ray diffraction were obtained.

### Refinement

All H atoms were placed in idealized positions (O—H = 0.85 Å, N—H = 0.86 Å and C—H = 0.95 Å) and refined as riding atoms with *U*_iso_(H) = 1.2*U*_eq_(C,N) and *U*_iso_(H) = 1.5*U*_eq_(O).

### Crystal data

**Table:**

C_16_H_12_N_6_S_2_·2H_2_O	*F*(000) = 808
*M**_r_* = 388.47	*D*_x_ = 1.417 Mg m^−^^3^
Monoclinic, *C*2/*c*	Mo *K*α radiation, λ = 0.71073 Å
Hall symbol: -C 2yc	Cell parameters from 3134 reflections
*a* = 12.3911 (13) Å	θ = 2.7–26.4°
*b* = 14.7125 (16) Å	µ = 0.32 mm^−^^1^
*c* = 10.2966 (11) Å	*T* = 293 K
β = 104.125 (2)°	Polyhedron, yellow
*V* = 1820.4 (3) Å^3^	0.40 × 0.20 × 0.18 mm
*Z* = 4	

### Data collection

**Table:**

Bruker SMART APEX CCD diffractometer	1953 independent reflections
Radiation source: fine-focus sealed tube	1679 reflections with *I* > 2σ(*I*)
graphite	*R*_int_ = 0.023
ω scans	θ_max_ = 27.0°, θ_min_ = 2.2°
Absorption correction: multi-scan (*SADABS*; Sheldrick, 1996)	*h* = −15→15
*T*_min_ = 0.884, *T*_max_ = 0.945	*k* = −18→18
7210 measured reflections	*l* = −13→13

### Refinement

**Table:**

Refinement on *F*^2^	Primary atom site location: structure-invariant direct methods
Least-squares matrix: full	Secondary atom site location: difference Fourier map
*R*[*F*^2^ > 2σ(*F*^2^)] = 0.042	Hydrogen site location: inferred from neighbouring sites
*wR*(*F*^2^) = 0.120	H-atom parameters constrained
*S* = 1.06	*w* = 1/[σ^2^(*F*_o_^2^) + (0.0691*P*)^2^ + 0.5857*P*] where *P* = (*F*_o_^2^ + 2*F*_c_^2^)/3
1953 reflections	(Δ/σ)_max_ < 0.001
118 parameters	Δρ_max_ = 0.20 e Å^−^^3^
0 restraints	Δρ_min_ = −0.18 e Å^−^^3^

### Special details

**Table:**

Geometry. All e.s.d.'s (except the e.s.d. in the dihedral angle between two l.s. planes) are estimated using the full covariance matrix. The cell e.s.d.'s are taken into account individually in the estimation of e.s.d.'s in distances, angles and torsion angles; correlations between e.s.d.'s in cell parameters are only used when they are defined by crystal symmetry. An approximate (isotropic) treatment of cell e.s.d.'s is used for estimating e.s.d.'s involving l.s. planes.
Refinement. Refinement of *F*^2^ against ALL reflections. The weighted *R*-factor *wR* and goodness of fit *S* are based on *F*^2^, conventional *R*-factors *R* are based on *F*, with *F* set to zero for negative *F*^2^. The threshold expression of *F*^2^ > σ(*F*^2^) is used only for calculating *R*-factors(gt) *etc*. and is not relevant to the choice of reflections for refinement. *R*-factors based on *F*^2^ are statistically about twice as large as those based on *F*, and *R*-factors based on ALL data will be even larger.

### Fractional atomic coordinates and isotropic or equivalent isotropic displacement parameters (Å^2^)

**Table:**

	*x*	*y*	*z*	*U*_iso_*/*U*_eq_	
S1	0.43278 (4)	0.48004 (3)	0.16853 (5)	0.0605 (2)	
N1	0.26234 (13)	0.68205 (9)	0.22572 (14)	0.0535 (4)	
H1B	0.2373	0.7366	0.2112	0.064*	
N2	0.33478 (13)	0.64316 (9)	0.16425 (15)	0.0554 (4)	
N3	0.28640 (12)	0.54543 (9)	0.30878 (14)	0.0501 (3)	
C1	0.13764 (18)	0.58140 (14)	0.4862 (2)	0.0673 (5)	
H1A	0.1698	0.5240	0.4897	0.081*	
C2	0.0682 (2)	0.60203 (17)	0.5693 (2)	0.0792 (6)	
H2A	0.0543	0.5586	0.6288	0.095*	
C3	0.01979 (18)	0.68669 (16)	0.5640 (2)	0.0729 (6)	
H3A	−0.0284	0.6999	0.6180	0.087*	
C4	0.0428 (2)	0.75119 (16)	0.4792 (2)	0.0723 (6)	
H4A	0.0115	0.8088	0.4773	0.087*	
C5	0.11194 (17)	0.73153 (13)	0.3963 (2)	0.0615 (5)	
H5A	0.1270	0.7759	0.3388	0.074*	
C6	0.15919 (13)	0.64584 (11)	0.39834 (16)	0.0478 (4)	
C7	0.23446 (14)	0.62407 (11)	0.31284 (16)	0.0461 (4)	
C8	0.34687 (14)	0.56106 (11)	0.21779 (17)	0.0503 (4)	
O1	0.27936 (14)	0.35247 (9)	0.37303 (13)	0.0762 (5)	
H1D	0.3018	0.3501	0.4572	0.091*	
H1C	0.2833	0.4118	0.3533	0.091*	

### Atomic displacement parameters (Å^2^)

**Table:**

	*U*^11^	*U*^22^	*U*^33^	*U*^12^	*U*^13^	*U*^23^
S1	0.0728 (4)	0.0462 (3)	0.0721 (3)	−0.00574 (19)	0.0364 (3)	−0.01332 (19)
N1	0.0701 (9)	0.0405 (7)	0.0563 (8)	0.0046 (6)	0.0276 (7)	0.0048 (6)
N2	0.0731 (9)	0.0448 (8)	0.0561 (8)	−0.0012 (7)	0.0305 (7)	0.0014 (6)
N3	0.0584 (8)	0.0412 (7)	0.0564 (8)	−0.0014 (6)	0.0248 (6)	0.0027 (6)
C1	0.0780 (13)	0.0589 (11)	0.0745 (12)	0.0101 (9)	0.0371 (10)	0.0135 (9)
C2	0.0917 (16)	0.0835 (15)	0.0757 (14)	0.0031 (12)	0.0463 (12)	0.0154 (11)
C3	0.0697 (13)	0.0864 (15)	0.0716 (13)	0.0043 (11)	0.0347 (10)	−0.0062 (11)
C4	0.0757 (13)	0.0681 (13)	0.0813 (14)	0.0161 (10)	0.0347 (11)	−0.0006 (10)
C5	0.0693 (12)	0.0539 (10)	0.0671 (11)	0.0098 (9)	0.0278 (9)	0.0082 (8)
C6	0.0478 (8)	0.0494 (9)	0.0477 (8)	−0.0011 (7)	0.0142 (7)	0.0003 (7)
C7	0.0517 (9)	0.0405 (8)	0.0476 (8)	−0.0031 (6)	0.0150 (7)	0.0014 (6)
C8	0.0596 (10)	0.0420 (8)	0.0543 (9)	−0.0052 (7)	0.0233 (7)	−0.0033 (7)
O1	0.1323 (14)	0.0421 (7)	0.0576 (8)	−0.0052 (7)	0.0300 (8)	−0.0033 (5)

### Geometric parameters (Å, °)

**Table:**

S1—C8	1.7536 (17)	C2—C3	1.378 (3)
S1—S1^i^	2.0556 (11)	C2—H2A	0.9300
N1—C7	1.343 (2)	C3—C4	1.366 (3)
N1—N2	1.346 (2)	C3—H3A	0.9300
N1—H1B	0.8600	C4—C5	1.380 (3)
N2—C8	1.321 (2)	C4—H4A	0.9300
N3—C7	1.330 (2)	C5—C6	1.388 (2)
N3—C8	1.355 (2)	C5—H5A	0.9300
C1—C6	1.381 (2)	C6—C7	1.466 (2)
C1—C2	1.386 (3)	O1—H1D	0.8434
C1—H1A	0.9300	O1—H1C	0.9007
			
C8—S1—S1^i^	101.08 (6)	C3—C4—H4A	119.7
C7—N1—N2	110.73 (14)	C5—C4—H4A	119.7
C7—N1—H1B	124.6	C4—C5—C6	120.28 (19)
N2—N1—H1B	124.6	C4—C5—H5A	119.9
C8—N2—N1	102.31 (13)	C6—C5—H5A	119.9
C7—N3—C8	103.16 (14)	C1—C6—C5	119.04 (17)
C6—C1—C2	120.15 (19)	C1—C6—C7	119.78 (16)
C6—C1—H1A	119.9	C5—C6—C7	121.14 (16)
C2—C1—H1A	119.9	N3—C7—N1	109.05 (14)
C3—C2—C1	120.2 (2)	N3—C7—C6	126.37 (15)
C3—C2—H2A	119.9	N1—C7—C6	124.58 (15)
C1—C2—H2A	119.9	N2—C8—N3	114.73 (15)
C4—C3—C2	119.78 (19)	N2—C8—S1	121.06 (13)
C4—C3—H3A	120.1	N3—C8—S1	124.19 (13)
C2—C3—H3A	120.1	H1D—O1—H1C	104.4
C3—C4—C5	120.5 (2)		

Symmetry codes: (i) −*x*+1, *y*, −*z*+1/2.

### Hydrogen-bond geometry (Å, °)

**Table:**

*D*—H···*A*	*D*—H	H···*A*	*D*···*A*	*D*—H···*A*
O1—H1C···N3	0.90	2.02	2.9210 (19)	178.
N1—H1B···O1^ii^	0.86	1.90	2.7077 (19)	156.
O1—H1D···N2^iii^	0.84	2.07	2.909 (2)	171.

Symmetry codes: (ii) −*x*+1/2, *y*+1/2, −*z*+1/2; (iii) *x*, −*y*+1, *z*+1/2.
